# The Effect of Visual Distinctiveness on Multiple Object Tracking Performance

**DOI:** 10.3389/fpsyg.2012.00307

**Published:** 2012-08-27

**Authors:** Piers D. L. Howe, Alex O. Holcombe

**Affiliations:** ^1^School of Psychological Sciences, University of MelbourneParkville, VIC, Australia; ^2^Department of Psychology, University of SydneySydney, NSW, Australia

**Keywords:** object tracking, MOT, feature, distinctiveness, visual search

## Abstract

Observers often need to attentively track moving objects. In everyday life, such objects are often visually distinctive. Previous studies have shown that tracking accuracy is increased when the targets contain a visual feature (e.g., a color) not possessed by the distractors. Conversely, a gain in tracking accuracy was not observed when the targets differed from the distractors by only a conjunction of features (Makovski and Jiang, 2009a). In this study we confirm that some conjunction targets have relatively little effect on tracking accuracy, but show that other conjunction targets can significantly aid tracking. For example, tracking accuracy is relatively high when the targets are small red squares and half the distractors are large red squares while the remaining distractors are small green squares. This seems to occur because the targets have a set of features (small and red) not shared by any one distractor. Attending to these features directs attention more to the targets than the distractors, thereby making the targets easier to track. Existing theories of attentive tracking cannot explain these results.

## Introduction

How does an observer make sense of an environment in which objects are constantly changing locations? One important strategy is to keep track of the locations of a handful of the most important objects. In this article we study this tracking ability and investigate to what extent visual distinctiveness can assist object tracking.

**Figure 1:**
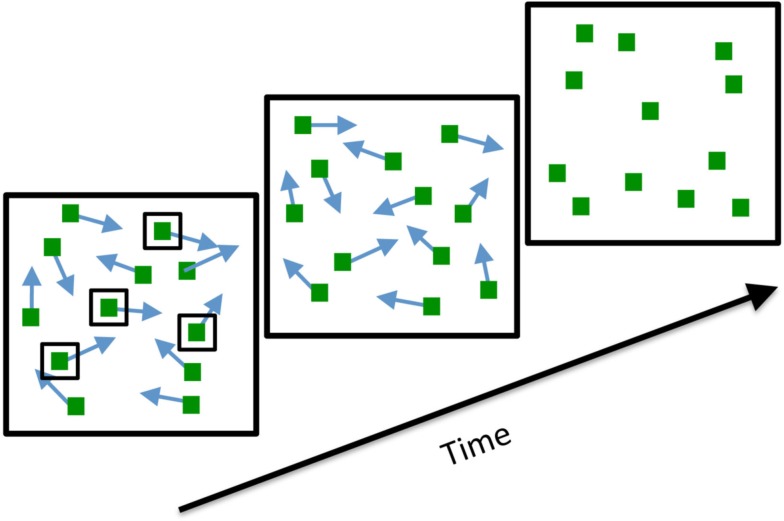
**The stimulus sequence used in Experiment 1**. Observers viewed a number of moving squares, a subset of which was briefly highlighted to indicate the targets to be tracked. At the end of the trial, all the squares stopped moving and the observer used the mouse to select all the targets.

The ability of humans to keep track of moving objects has typically been studied using the multiple object tracking (MOT) paradigm (Pylyshyn and Storm, 1988). In a typical example of this paradigm, an observer views a number of identical objects that move about a computer monitor (Figure 1). At the start of the trial, some of the objects are briefly ringed to indicate that these are the targets to be tracked. The rings disappear and the objects continue to move for several more seconds before finally coming to a halt. The observer is then asked to identify the targets. Despite its simplicity, this paradigm has taught us a great deal about what limits our ability to attentively track multiple moving objects (Scholl, 2001, 2009; Cavanagh and Alvarez, 2005).

A potential drawback with this paradigm is that during the movement phase all the objects are identical. Conversely, in an everyday setting, the identities of the target objects are typically continuously visible while the observer tracks them. Horowitz et al. (2007, Experiment 5) investigated whether the task was made easier by having the object identities continuously visible during the tracking phase. In one condition, the objects were all unique pictures of cartoon animals. At the start of the trial, a subset of the objects were briefly flashed to indicate that these were the targets to be tracked. During the trial, all the objects then moved about the computer monitor, periodically hiding behind pictures of cactuses. Each trial was timed to end when all the objects were simultaneously occluded. The observer was then asked to identify the locations of all the targets. A second condition was identical to the first, except that all the objects were identical. On average, observers could track 3.1 items in the unique condition, but only 2.5 items in the identical condition, thereby demonstrating that object distinctiveness can aid tracking.

From the Horowitz et al. (2007) study it is unclear how object distinctiveness aids tracking. One possibility is that it aids only target recovery. According to this account, distinctive targets are just a likely to be lost as non-distinctive targets, but the former can more readily be recovered during the tracking period. Every time a target is lost during the tracking phase, the observer visually searches for it. The more distinctive the target, the easier it is to recover, causing tracking accuracy to be higher for distinctive targets than for non-distinctive targets. Makovski and Jiang (2009b) ran a number of experiments to investigate whether target recovery is the root cause of the distinctiveness advantage.

In the *all unique* condition of Makovski and Jiang (2009b) the tracking display contained eight disks, each one a unique color. Observers were required to keep track of the locations of four target disks (e.g., the red one, the green one, the blue one, and the yellow one). At the end of the trial, the disks turned black and stopped moving. The observers were then required to identify the four targets, but were not required to distinguish between them. Tracking accuracy was substantially higher in the *all unique* condition than in the *homogeneous* condition where all the disks were the same color, thereby replicating the distinctiveness advantage observed by Horowitz et al. (2007). However, tracking accuracy in the *all unique* condition was substantially reduced if all the targets did not move in a smooth manner. In this condition, halfway through the trial all the disks suddenly underwent a discontinuous jump, and tracking accuracy dropped from 73 to 58% where the chance level of accuracy was 50%. If the distinctiveness advantage was primarily due to a target recovery strategy, observers should have had little difficulty in recovering the targets after the jump.

In a second experiment, Makovski and Jiang (2009b) measured tracking accuracy in the *all unique* condition as a function of the minimum target-distractor distance. It was expected that if observers were employing a search strategy, they would be able to recover from situations where a target passed close to a distractor, so tracking accuracy should not vary with the minimum target-distractor distance. Contrary to this, the closer the targets were allowed to pass the distractors, the worse the tracking accuracy. Together, these two experiments suggest that target recovery does not make a major contribution to the distinctiveness advantage, so for the purposes of this study we will assume that the distinctiveness advantage is primarily caused by some other mechanism.

The aspects of object distinctiveness that benefit tracking are not understood. Makovski and Jiang (2009a) found that when targets differ from distractors in a feature such as color or shape, tracking performance benefits. They also tested a situation where targets shared the features of the distractors but differed in the combination (conjunction) of features. They concluded that distinctiveness of conjunctions does not benefit performance, only distinctiveness of features.

In the Makovski and Jiang (2009a) experiment the observers viewed a number of colored digits that moved about a computer screen. In the *homogeneous* condition, all the digits were identical. In the *color-distinct* condition, each digit was a different color. In the *digit-distinct* condition, each digit was different. In the *conjunction-distinct* condition, the targets and distractors shared the same colors and digit identities but no target was identical to any distractor. For example, if one of the targets was a blue “2,” then one of the distractors would be blue, another distractor would be a “2,” but none of the distractors would be both blue and a “2.” In addition, the targets were all different from each other – each was a different color and a different digit. Tracking accuracy was significantly greater in the *color-distinct* and *digit-distinct* conditions than in the *homogeneous* condition, confirming the finding of Makovski and Jiang (2009b) that if the targets contained visual features not shared by the distractors then tracking accuracy is improved relative to the *homogenous* condition. Conversely, tracking accuracy in the *conjunction-distinct* condition was not better than that in the *homogeneous* condition. In summary, Makovski and Jiang (2009a) found that featural distinctiveness, but not conjunction distinctiveness, enhances tracking performance.

It may be premature to conclude that having targets differ from distractors by their feature conjunction cannot benefit performance. In the *conjunction-distinct* condition of Makovski and Jiang (2009a), each target was defined by a different combination of features. If instead the same conjunction is used for all the targets, attending to the two features of that conjunction should lead to preferential processing of the targets over the distractors, provided that no one distractor contains the same two features. This has not been tested in tracking, but previous evidence indicates that it benefits visual search performance (Wolfe et al., 1989). Indeed, this was key to the formulation of the Guided Search Model of visual search (Wolfe et al., 1989). In acknowledgment of this we will refer to situations with such target-distractor relationships as “guidable.” Other display arrangements with conjunction targets might result in “non-guidable” situations.

We suggest that the *conjunction-distinct* condition used by Makovski and Jiang (2009a) resulted in non-guidable targets. Each target was a distinct conjunction of features and taken together the targets had exactly the same features as the distractors. Therefore biasing attention toward the target features would direct attention just as much to the distractors as to the targets.

Conversely, in our novel tracking condition, which we will call the *size-color conjunction* condition, the targets were guidable. We used targets that were all identical and defined by a unique combination of two features, color and size, only one of which was shared with any one distractor. Thus, some of the distractors would be the same size as the targets while other distractors would be same color as the targets, but no distractor would be both the same color and size as the targets. For example in one trial the targets could be small red squares and the distractors could be large red squares and small green squares. Attending to the features *small* and *red* would direct attention more to the targets than to the distractors. We suggest that this should make the targets easier to track. The possibility for featural attention to provide a benefit arises because the targets contain two features, in this case *small* and *red*, only one of which is shared with any one distractor.

When participants attend to a *size-color conjunction* target, we propose that their attention becomes globally biased toward the target features. Thanks to top-down feedback in the visual cortex, such feature-based attention increases the activity of the neurons that prefer the targeted features throughout the visual field (Treue and Martinez Trujillo, 1999; Bichot et al., 2005; Maunsell and Treue, 2006; Serences and Boyton, 2007; David et al., 2008). Thus, because the targets contains a unique set of features (e.g., *small* and *red)* not shared by any one distractor, attending to the target features increases the neural activation of the target-responding neurons relative to the neural activation of the distractor-responding neurons. During tracking, an important possible consequence is that in situations where the target might be confused with a distractor if the objects were identical, this greater neural activation of the target-responding neurons prevents the loss of the target.

Although it is already known that this type of target-distractor relationship benefits visual search (Wolfe et al., 1989), it is unknown whether the feature-based attention involved can also be applied during a tracking task to benefit performance.

**Figure 2:**
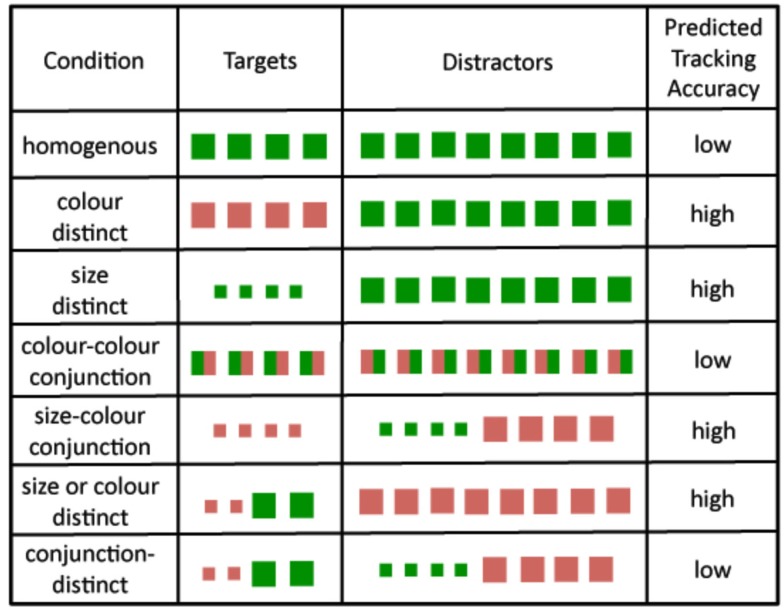
**The seven conditions of Experiment 1**. The colors and sizes were randomly assigned, although here only one particular combination is shown. Please see the text for more details.

A non-guidable situation can be arranged even when all the targets are identical. For example, suppose the targets were squares whose left halves were green and whose right halves were red. Further suppose that the left halves of the distractors were red and the right halves were green (see *color–color conjunction*; Figure 2). Attending to the features “red” and “green” would direct attention equally to both the targets and the distractors. Since attention would not be directed preferentially to the targets, we term this condition as non-guidable. We predict that non-guidable situations should not lead to an improvement in tracking accuracy relative to the condition where all the objects are identical.

The aim of our study was to test the claim that conjunction distinctiveness can enhance tracking performance, contrary to what has previously been suggested (Makovski and Jiang, 2009a). Specifically, we investigated whether tracking accuracy is increased when the targets are guidable, i.e., attending to the target features will direct attention more to the targets than to the distractors. To preview our results, we found evidence that supports this hypothesis.

## Experiment 1

### Method

#### Participants

There were 15 participants (age range 21–41, six male). They all reported normal or corrected-to-normal visual acuity and reported not being colorblind. All observers provided informed written consent and the study was approved by the Departmental Human Ethics Advisory Group in the School of Psychological Sciences at the University of Melbourne.

#### Apparatus and stimuli

Subjects viewed a 21″ CRT monitor at a resolution of 1280 × 1024 pixels with a 85 Hz frame rate from a distance of approximately 60 cm. Stimuli were presented in MATLAB (Mathworks, Natick, MA, USA) using Psychophysics toolbox (Brainard, 1997; Pelli, 1997).

The stimuli for the seven conditions are shown in Figure 2. In each condition the observer saw 12 squares. Depending on the condition, the squares could be large, 0.75° × 0.75° of visual angle (°), or small, 0.375° × 0.375°. Similarly, they could be one of four colors: red, green, yellow, or blue.

In the *homogeneous* condition all the squares were the same size and had the same randomly chosen color. This condition provided a baseline of performance to compare to the other conditions.

In the *color-distinct* condition, the targets and the distractors were both 0.75° × 0.75° but the targets were one color and the distractors were a different color, with both colors randomly chosen from the set of four possible colors, with the constraint that they could not both be the same. In the *size-distinct* condition the targets and distractors were different sizes, with the sizes chosen randomly (i.e., either 0.75° × 0.75° or 0.375° × 0.375°). In both these conditions the targets had a feature not shared with the distractors. The purpose of these two conditions was to test the previous finding that featural distinctiveness enhances tracking accuracy relative to the homogenous condition (Makovski and Jiang, 2009a,b).

In the *color–color conjunction* all the squares were 0.75° × 0.75°, bicolored and shared the same two colors. The left-right arrangement of the colors was reversed for the targets and the distractors. For example, if the left side of the targets was green and the right side was red then the left side of the distractors would be red and the right side would be green. Thus the targets and the distractors shared the same features (i.e., they shared the same two colors). The point of this condition was to confirm that non-guidable targets do not enhance tracking accuracy relative to the homogenous condition.

In the *size-color conjunction* condition the targets contained a single combination of features not shared by any one distractor. For example, the targets could be small red squares and the distractors could be small green squares and large red squares. Alternatively, the targets might be large blue squares and the distractors could be small blue squares and large yellow squares. All possible combinations of sizes and colors were equally likely to be tested subject to the constraint that the targets always contained a unique set of features not shared by any one distractor. Thus, the targets were always guidable. The point of this condition was to test our hypothesis that conjunction targets enhance tracking accuracy relative to the homogenous condition only when the targets are guidable, i.e., attending to the target features will direct attention preferentially to the targets as opposed to the distractors. (In contrast, in the *conjunction-distinct* condition of Makovski and Jiang, 2009a the targets were non-guidable.) In particular, we predicted that tracking accuracy in the *size-color conjunction* condition would be much greater than that in the *color–color conjunction* condition where the targets did not have a unique set of features not shared by the distractors, so were also not guidable.

An important prediction of our feature-based attention hypothesis is that it should be possible to preferentially direct attention to the targets, even when the targets are not all the same. The *size or color-distinct* condition tested this prediction. The targets were not homogenous: two were one size and color and two were a different size and color. In addition, they differed from the distractors either in size or in color, but not both. All possible combinations of size and color and were equally likely to be tested subject to the constraint that (a) all the distractors were always the same size and color, (b) half the targets had the same color as the distractors but were a different size, and (c) the remaining targets were the same size as the distractors but were a different color. For example, two targets might be small red squares and the other two targets might be large green squares. In this case, the distractors might be all large red squares. Because the targets contained a unique set of features (e.g., small and green) not shared by any one distractor, the feature-based attention hypothesis predicts that by attending to these two features, attention could be directed preferentially to the targets, thereby increasing tracking accuracy relative to the *homogenous* condition.

Finally, the *conjunction-distinct* condition tested a corollary prediction. It used exactly the same target set as the *size* or *color-distinct* condition (i.e., half the targets were one color and size and the remaining targets were a different color and size). The crucial difference was that the distractors were no longer homogenous and taken as a set contained the same set of features as the targets, though arranged differently so that no distractor was identical to any target. Half the distractors were always one color and size and the remaining distractors were always a different color and size. For example, two targets might be small red squares and the other two targets might be large green squares. In this case, half the distractors would be small green squares and the remainder would be large red squares. Since the targets and the distractors both contained the same four features (in this example: small, large, green, and red), the feature-based attention hypothesis would not predict tracking accuracy to be greater in this condition than in the *homogenous* condition. Consistent with this prediction, Makovski and Jiang (2009a) found that tracking accuracy in their *conjunction-distinct* condition was not better than in their *homogenous* condition. Indeed, it was slightly worse, suggesting that observers would have done better by ignoring the differences between the targets and the distractors.

#### Procedure

Each trial lasted a random duration between 3 and 10 s, during which the squares moved continuously. For the first 2 s, the targets were surrounded by black rings to identify them. At the end of the trial, multi-color masks covered the squares for 0.25 s. These masks subtended 0.75° × 0.75° and comprised the four colors randomly arranged for each trial in a concentric fashion (i.e., each mask comprised four concentric rings, with each ring being a different color). During the masking, all the squares became white, so that after the masks were removed they were all identical. The observer then used a computer mouse to click on all four targets. The observer did not have to indicate which target had which attributes, so did not have to distinguish between the targets. After the trial ended the observer was informed how many targets he/she got correct. Accuracy was defined as the average proportion of correctly identified targets. As 4 of the 12 squares were targets, chance accuracy was 33%. The experiment began with 10 practice trials followed by 280 test trials, evenly divided between the seven conditions, presented in a random order.

### Results and discussion

**Figure 3:**
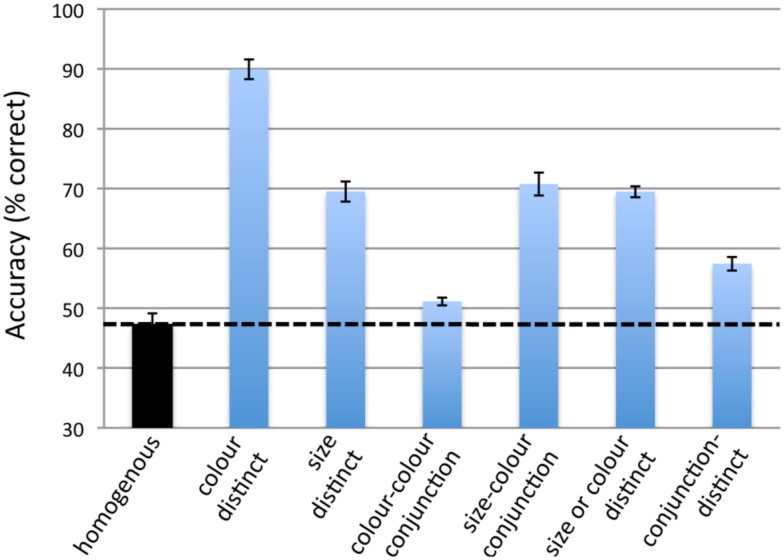
**The results of Experiment 1**. Error bars represent repeated measures standard error (Morey, 2008). Chance performance is at 33%. The dotted line represents the performance in the homogeneous condition.

The results are shown in Figure 3. A repeated measures ANOVA with a Greenhouse–Geisser correction found a significant effect of condition *F*(2.46, 34.5) = 111, *p* < 0.001. We performed planned repeated measures *t*-tests to test the above predictions.

As expected, the two conditions where the targets were featurally distinctive yielded greater tracking accuracy than the homogenous condition: for the *color-distinct* condition *t*(14) = 25.0, *p* < 0.001 and for the *size-distinct* condition *t*(14) = 7.58, *p* < 0.001. These results confirm previous findings that featural distinctiveness aids tracking (Makovski and Jiang, 2009a,b).

As predicted by the feature-based attention hypothesis, accuracy in the *size-color conjunction* condition was also greater than that in the *homogenous* condition [*t*(14) = 8.18, *p* < 0.001] and that in the *color–color conjunction* condition [*t*(14) = 9.80, *p* < 0.001]. Accuracy in the *color–color conjunction* condition was slightly greater than that in the *homogeneous* condition [*t*(14) = 2.52, *p* = 0.024], though this difference was very small (51 versus 47%). This last result is somewhat surprising as the feature-based attention hypothesis would predict the two conditions to be equal.

As predicted by the feature-based attention hypothesis, accuracy in the *size* or *color-distinct* condition was also significantly greater than that in the *homogeneous* condition [*t*(14) = 11.5, *p* < 0.001] and that in the *conjunction-distinct* condition [*t*(14) = 8.85, *p* < 0.001].

Surprisingly, tracking accuracy in the *conjunction-distinct* condition was slightly greater than that in the *homogeneous* condition [*t*(14) = 4.98, *p* < 0.001] – 57 versus 47%. This final result is not predicted by the feature-based attention hypothesis but could explained by postulating that target recovery provides a small benefit to tracking accuracy, as detailed in the Section “Discussion.” In summary, these results are generally consistent with the feature-based attention hypothesis but the final result suggests that a secondary mechanism also contributes to tracking accuracy.

## Experiment 2

**Figure 4:**
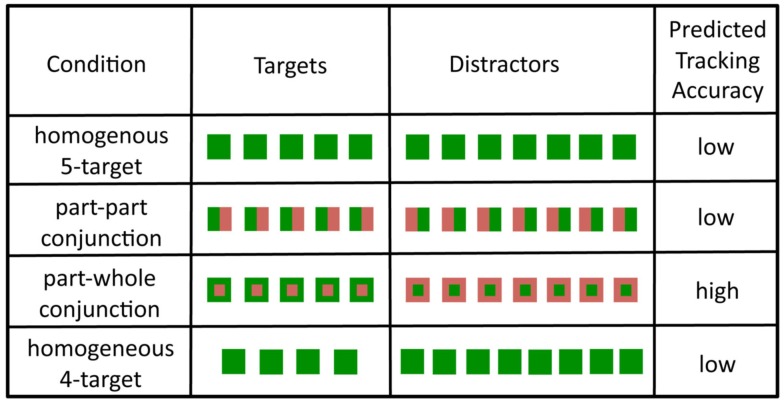
**The stimulus conditions used in Experiment 2**. The colors were randomly assigned, although here only one particular combination is shown. Please see the text for more details.

The color–color conjunction tested in Experiment 1 is known as a part–part conjunction as each square was divided into two equal parts and each part was a different color (Wolfe et al., 1994). An alternative arrangement is a part-whole color conjunction, for example a green square with a red center, as shown in Figure 4. Whereas part–part color conjunctions lead to very inefficient visual search, part-whole color conjunctions can be quite efficient, with search times that increase only moderately as the number of items in the search display increases (Wolfe et al., 1994). This has led to the claim that feature attention can be directed to *part* and *whole* components separately (Wolfe et al., 1994). Thus, tracking accuracy should be greater for part-whole conjunction targets than for part–part conjunction targets. For example, when tracking targets that are green squares with red centers among distractors that are red squares with green centers, the observer should be able to direct attention to “green whole” and “red part.” Consequently, it should be possible for featural attention to preferentially bias the targets, as opposed to the distractors, thereby making the targets easier to track than in the *homogeneous* condition. Experiment 2 tested this prediction and found it to hold.

Although we have offered an explanation for this finding in terms of the feature-based attention hypothesis, other explanations are potentially possible. For example, it could be that target distinctiveness does not facilitate tracking *per se* but instead allows the observers not to have to track all the targets. For example, when asked to track five targets, observers might instead choose just to track four of them and to continuously search for the fifth. We shall refer to this as the track-and-search strategy. Because this explanation assumes that tracking distinctiveness does not enhance tracking *per se* it predicts that tracking five distinctive targets is as hard as tracking four non-distinctive targets while performing visual search for a fifth. Thus, tracking accuracy for five distinctive targets should not be greater than that for tracking four non-distinctive targets. Experiment 2 tested this prediction and found it not to be correct, suggesting that this particular search-and-track strategy is not used by observers.

### Method

There were 15 participants (age range 21–34, five male). The method of this experiment was similar to that of the previous experiment, with a multi-colored mask covering the objects at the end of the trial, after which the squares reappeared in white, so that they were all identical. The difference from Experiment 1 was that at the end of each trial one item was ringed and the participant was asked only whether or not the ringed item was a target. Conversely, in Experiment 1 participants were asked to click on all the targets. Only asking one question per condition allowed us to compare conditions with different numbers of targets in an unbiased fashion. At the end of each trial the observer was informed whether he/she was correct or not.

In total, there were four conditions (Figure 4). There was a *part–part color conjunction* condition and the *part-whole color conjunction* condition. In both of these conditions the observer was required to track five targets. There were also two homogeneous conditions in which all objects were uniform in color and size. In one, the observer tracked five targets and in the other the observer tracked four targets. The experiment started with 10 practice trials and then there were 200 trials, equally divided between the four conditions.

### Results and discussion

**Figure 5:**
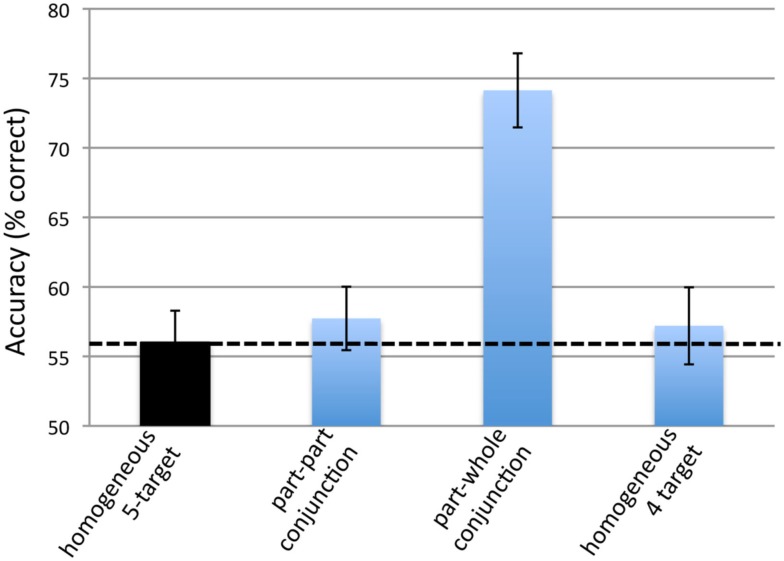
**The results from Experiment 2**. Error bars represent repeated measures standard error (Morey, 2008). Chance performance is at 50%. The dotted line represents performance in the homogeneous condition.

The feature-based attention hypothesis predicts that accuracy should be greater in the *part-whole* conjunction condition than in both the *part–part conjunction* condition and in the *homogenous 5-target* condition. As shown by Figure 5, these predictions were found to be true.

Tracking accuracy in each of the four conditions was significantly greater than chance: for the *homogenous 5-target* condition, *t*(14) = 3.67, *p* = 0.003. For the *part–part conjunction* condition, *t*(14) = 3.08, *p* = 0.008. For the *part-whole conjunction* condition, *t*(14) = 7.16, *p* < 0.001. For the *homogenous 4-target* condition, *t*(14) = 2.31, *p* = 0.037.

A repeated measures ANOVA found a significant effect of condition [*F*(3,42) = 15.7, *p* < 0.001]. We performed planned repeated measures *t*-tests to test the predictions described in the Section “Introduction.” Accuracy in the *part–part conjunction* condition was again slightly greater than that in the *homogeneous 5-target* condition, but this difference was not significant [*t*(14) = 0.696, *p* = 0.498]. This result is in keeping with our finding from Experiment 1 that accuracy in these two conditions are very similar, showing that part–part color conjunctions do not substantially aid tracking. Conversely, accuracy in the *part-whole color conjunction* condition was significantly greater than that in the *homogeneous 5-target* condition [*t*(14) = 6.12, *p* < 0.001] and that of the *part–part color conjunction* condition [*t*(14) = 5.10, *p* < 0.001]. This is in keeping with the prediction of the feature-based attention hypothesis that part-whole conjunctions should aid tracking. To put in perspective how much part-whole conjunctions can aid tracking we compared accuracy in this condition to that in the *homogeneous 4-target* condition. Accuracy was significantly greater in the *part-whole color conjunction* condition [*t*(14) = 5.09, *p* < 0.001], even though in this condition observers were required to track one additional target. This finding argues against the track-and-search hypothesis described previously.

## Discussion

In Experiment 1 we considered two conditions where the targets contained a single feature not shared by any of the distractors: the *size-distinct* condition and the *color-distinct* condition. Consistent with Makovski and Jiang (2009b), tracking accuracy was much greater in both conditions than in the *homogeneous* condition where all the disks were identical, thereby confirming that featural distinctiveness can aid tracking.

We then considered two conjunction conditions. In one of them, the *size-color conjunction* condition, the targets were all the same and contained a unique set of features not shared by any one distractor. These targets were therefore guidable in that attending to the target features would direct attention more to the targets than to the distractors. In the other conjunction condition, the *color–color conjunction* condition, each distractor had the same two features as each target, making the targets non-guidable. Consistent with our feature-based attention hypothesis, tracking accuracy was much higher in the guidable situation (i.e., the *size-color conjunction* condition) than in the non-guidable situation (i.e., *the color–color conjunction* condition) or in the *homogeneous* condition. This contradicts a previous conclusion that conjunction targets cannot increase tracking accuracy (Makovski and Jiang, 2009a).

An important prediction of our feature-based attention hypothesis is tracking enhancement should occur even when the targets are not identical. The *size* or *color-distinct* condition tested this prediction. In this condition, the targets were not all the same but all contained a feature not present in any of the distractors, so again were guidable. Thus, the feature-based attention hypothesis would predict that by attending to the target features attention could be preferentially directed toward the targets rather than the distractors, thereby making the targets easier to track. This prediction was found to be true.

A corollary prediction is that using distractors that contain the same set of features as the targets should cause the targets to be no longer guidable and therefore reduce tracking performance. This was tested in the *conjunction-distinct* condition where it was observed that tracking accuracy was indeed less than that in *size or color-distinct* condition.

The color–color conjunction used in Experiment 1 is known as a part–part conjunction. The targets were bicolored squares, the left side of which was one color and the right side a different color. Alternatively, one could construct a target where the center was one color and the surrounding was a different color. Such targets are known as part-whole conjunctions. Visual search results suggest that part and whole information can be attended separately (Wolfe et al., 1994). Consequently, part-whole conjunctions should aid tracking. For example, if the targets are green squares with red centers and the distractors are red squares with a green centers, attending to “green wholes” and “red centers” should direct attention preferentially to the targets, thereby increasing tracking accuracy. In other words, part-whole conjunction targets should be guidable. Experiment 2 verified that tracking accuracy was indeed greater for part-whole conjunctions than for part–part conjunctions or for targets that were identical to the distractors (i.e., the homogeneous condition).

The above results can all be accounted for by our feature-based attention hypothesis. However, there are some results that are not consistent with this hypothesis. For example, it cannot explain why in Experiment 1 tracking accuracy in the *conjunction-distinct* condition was (slightly) greater than that in the *homogenous* condition.

As explained in the Section “Introduction,” we had assumed that a target recovery strategy does not play a major role in tracking distinctive targets. We made this assumption because of the findings of Makovski and Jiang (2009b). However, those findings do not prove that target recovery plays *no* role in object tracking. Target recovery may play a minor role (Horowitz et al., 2007) and this would explain why tracking accuracy in the *conjunction-distinct* condition was slightly greater than that in the *homogenous* condition, because only in the former condition could the targets be recovered if they were lost.

No published theory of object tracking can explain our findings. Most theories of object tracking were designed to explain the standard multiple object paradigm where all the objects are identical. As such, these theories typically do not address target distinctiveness (Pylyshyn and Storm, 1988; Cavanagh and Alvarez, 2005; Alvarez and Franconeri, 2007; Franconeri et al., 2009, 2010). One exception is a theory proposed by Yantis (1992). It addresses target distinctiveness, at least implicitly. This theory proposes that targets are tracked by grouping them together to form a single visual object and that observers track this single visual object instead of tracking the individual targets. The grouping should be easier if the targets were all one color and the distractors were all a different color because then the targets would naturally segregate from the distractors. Thus, this theory could explain why in Experiment 1 tracking accuracy is better in the *color-distinct* condition than in the *homogenous* condition. However, it is not clear how this theory could explain why tracking accuracy in the *size or color-distinct* condition was greater than that in the *homogeneous* conditions because in the former condition the targets were not all the same, which presumably would have made it more difficult to perceptually group them together.

According to the model of multiple identity tracking (MOMIT; Oksama and Hyona, 2008), each target is attended to in turn and every time a target is attended its location is noted. When it is time to reattend a given target the object that is closest to that target’s previously remembered location is selected and its appearance is compared to the memory of that target. If it does not match, another object is selected and the process repeats until the target is recovered. Thus, MOMIT predicts that tracking accuracy should be greater when targets are distinctive than when they are not because in the former case the observer can recover from tracking errors. While this can explain our finding that tracking accuracy was greater in the *size-color conjunction* condition than in the *homogenous* condition in Experiment 1, it cannot explain why performance was so poor in the *color–color conjunction* condition. Indeed, because MOMIT does not distinguish between different types of conjunction targets it implies that performance in the *color–color conjunction* condition should be approximately equal to that in the *size-color conjunction* condition. In fact, performance was much greater in the latter condition than the former (71 versus 51%).

A model by Vul et al. (2009) is similar to MOMIT in that it assumes that targets are attended only periodically and that identity information can be used to solve the correspondence problem (i.e., to determine which objects at one point in time correspond to which objects at a previous point at time). Thus, like MOMIT, it would predict that performance would be better in the *size-color conjunction* condition than in the *homogeneous* condition, but it is also not clear how it could explain why performance is greater in the *size-color conjunction* condition than in the *color–color conjunction* condition.

Makovski and Jiang (2009a, p. 193) suggest that the fact that part–part color–color conjunction targets result in a relatively small increase in tracking accuracy shows that features are not properly conjoined during attentive tracking. In other words, the tracking system does not have access to bound feature representations so, for example, cannot distinguish between a red-green bicolored square from a green-red bicolored square. Our feature-based attention hypothesis is consistent with this explanation as it does not require binding to occur.

In conclusion, this is the first study to demonstrate that conjunction targets can increase tracking accuracy. Our findings indicate that this advantage is greatest when all the conjunction targets contain the same combination of features not shared by any one distractor. However, even when this condition is not satisfied, conjunction targets can still aid tracking, albeit to a lesser extent. Our results do not imply that the attentional tracking system has access to bound target representations and instead we have argued for a feature-based attention account.

## Conflict of Interest Statement

The authors declare that the research was conducted in the absence of any commercial or financial relationships that could be construed as a potential conflict of interest.
